# Ontogenetic similarities between giraffe and sauropod neck osteological mobility

**DOI:** 10.1371/journal.pone.0227537

**Published:** 2020-01-13

**Authors:** Daniel Vidal, Pedro Mocho, Adrián Páramo, José Luis Sanz, Francisco Ortega

**Affiliations:** 1 Grupo de Biología Evolutiva, Facultad de Ciencias, UNED, Paseo Senda Del Rey, Madrid, Spain; 2 Instituto Dom Luiz, Universidade de Lisboa, Bloco C6, 38 Piso, sala 6.3.57, Campo Grande, Lisbon, Portugal; 3 The Dinosaur Institute, Natural History Museum of Los Angeles County, Los Angeles, CA, United States of America; 4 Unidad de Paleontología, Facultad de Ciencias, Universidad Autónoma de Madrid, Calle Darwin, Madrid, Spain; 5 Real Academia Española de Ciencias Exactas, Físicas y Naturales, Calle Valverde, Madrid, Spain; Universiteit Maastricht, NETHERLANDS

## Abstract

The functional morphology of sauropod dinosaur long necks has been studied extensively, with virtual approaches yielding results that are difficult to obtain with actual fossils, due to their extreme fragility and size. However, analyses on virtual fossils have been questioned on several of their premises, such as the ability to accurately reconstruct intervertebral tissue with only skeletal data; or whether zygapophyseal overlap can be used to determine the limits of range of motion, since some extreme neck poses in extant giraffes have been claimed not to retain any zygapophyseal overlap. We compared articulation and range of motion in extant giraffes with the exceptionally well-preserved and complete basally branching eusauropod *Spinophorosaurus nigerensis* from the Middle (?) Jurassic of Niger, under the same virtual paleontology protocols. We examined the articulation and range of motion on grown and young specimens of both *Spinophorosaurus* and giraffes in order to record any potential changes during ontogeny. Also, the postures of virtual giraffes were compared with previously published data from living animals in the wild. Our analyses show that: (i) articulation of virtual bones in osteologically neutral pose (ONP) does enable accurate prediction of the amount of inter-vertebral space in giraffes and, roughly, in *Spinophorosaurus*; (ii) even the most extreme neck postures attained by living giraffes in the wild do not require to disarticulate cervical vertebrae; (iii) both living giraffes and *Spinophorosaurus* have large intervertebral spaces between their cervical centra in early ontogenetical stages, which decrease as ontogeny advances; and (iv) that grown specimens have a greater osteological range of motion in living giraffes and *Spinophorosaurus*.

## Introduction

The elongated neck of sauropod dinosaurs is one of their more notable features. These necks vary tremendously in length, both in relative and absolute terms: from the relatively short-necked *Brachytrachelopan mesai*, which has a one meter long neck representing less than a quarter of the length of its axial skeleton [1], to the up to nine meter neck of *Mamenchisaurus sp*., which accounts for approximately half the length of its axial skeleton [2,3]. The number of cervical vertebrae in sauropods also varies, with a basal condition of likely 12 cervical vertebrae, present in most sauropods with complete necks [4]. to up to 19 cervical vertebrae in *Mamenchisaurus hochuanensis* [2].

There has been a keen interest in understanding the functional capabilities of sauropod necks, particularly their mobility: while early works described these necks as extremely mobile, with even "avian-like flexibility" [5,6], sauropod neck mobility would not be rigorously analyzed until the late 1980s, when Martin published his analyses on the mobility of the Leicester "*Cetiosaurus"* specimen (LCM G468.1968). Using the actual fossils when assembling the skeleton, Martin found that this Middle Jurassic sauropod had a very limited range of motion, both in dorsoventral and lateral planes [7]. "*Cetiosaurus"* was found barely able to lift the skull 3.5 meters from the ground and make an arc of 4.5 meters in the lateral plane before disarticulation [7]. Later works of Stevens and Parrish, with their computerized DinoMorph models, also found diplodocid sauropods *Diplopdocus* and *Apatosaurus* to have not very flexible necks [8–10], with *Diplodocus* in particular barely able to lift its head above its shoulder-height [8].

However, some researchers have questioned the DinoMorph results, on the grounds of (i) an arbitrary 50% zygapophyseal safety limit for disarticulation, by which the pre- and postzygapophyseal facets would always retain at least a 50% overlap for any given posture [11], and (ii) an underestimation of the amount of intervertebral and zygapophyseal soft tissue/cartilage [12], which would make the necks have more range of motion in the vertical component [13]. Dzemski and Christian conducted range of motion analyses on three long-necked extant animals (*Struthio camelus*, *Camelus bactrianus* and *Giraffa camelopardalis*) by manipulating their bones manually, noticing that *in vivo* flexibility was attainable with the bare bones, although with less zygapophyseal overlap than 50% [11], which had been proposed by Stevens and Parrish as the limit for range of motion [8].

Research on the non-sauropod sauropodomorph *Plateosaurus* [14,15] was crucial in establishing high fidelity 3D-scanned almost complete skeletons as one of the optimal ways to build skeletal mounts, with several advantages over using real fossil bones [15]. Among those advantages is the ability to operate more than one element at one time without external supports, hence minimizing damage to the original fossils [15]. However, few sauropod taxa account for a completely known neck, and fewer are known by a complete neck from a single individual [16]. Overall, there are roughly above a dozen taxa with complete necks known from a single specimen, and, among those, few are well preserved or easily accessible (since the original fossils are exhibited as mounted skeletons). Therefore, very few range of motion analyses have been carried out on complete, well-preserved sauropod necks, and the osteological neck motion capabilities of most sauropods remain largely unknown.

**Fig 1:**
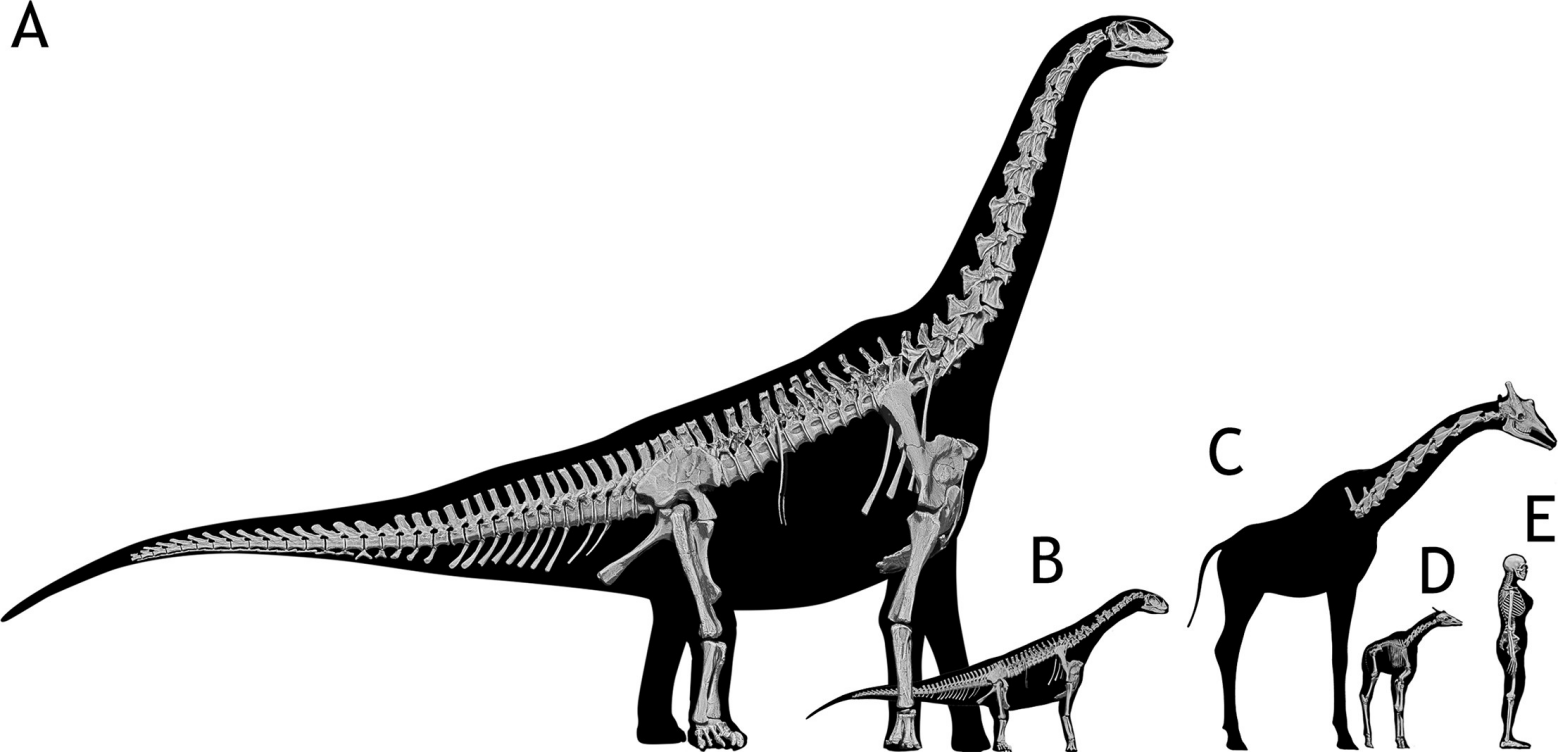
Size comparison of extant and extinct analyzed specimens. A—*Spinophorosaurus nigerensis* adult composite specimen. B—*Spinophorosaurus nigerensis* juvenile composite specimen. C—*Giraffa camelopardalis* adult specimen. D—*Giraffa camelopardalis* newborn specimen (TMM M-16050). E—1700 mm. *Homo sapiens* for scale.

*Spinophorosaurus nigerensis*, an early branching Eusauropod sauropod from the Middle? Jurassic of Niger [17,18], is one of the few sauropods known with an almost complete, well-preserved neck. Also, a putative juvenile *Spinophorosaurus* specimen was retrieved few meters away from the holotype [19], consisting of an almost complete, articulated cervical series and some dorsal vertebrae and rib remains. Since two almost complete necks are known for *Spinophorosaurus nigerensis* (Fig 1A and 1B), neck mobility can be tested minimizing reconstructive speculation in two sauropod specimens at two markedly different ontogenetic stages for the first time.

The following hypotheses have been evaluated: (i) osteology alone does not allow to estimate the amount of intervertebral space [20,21], (ii) some neck postures attained in life require disarticulating vertebrae [10,12], (iii) the amount of intervertebral space diminishes with ontogenetic development [12], and (iv) the range of motion of the neck increases with ontogenetic development. Given the size and fragility of the adult *Spinophorosaurus* skeleton and all the potential dangers of working with real fossils, a virtual paleontology approach was taken. Also, since both range of motion and intervertebral space is known to vary with ontogeny in extant *Giraffa camelopardalis*, we also analyzed two giraffe specimens at two different ontogenetic stages (adult and newborn, Fig 1). We followed the same methodology with both giraffes to assess whether the analyses with digital skeletons would render results compatible with *in vivo* observations and if that compatibility can help to interpret the obtained results from the analyses of the two necks referred to *Spinophorosaurus*.

## Material

**Fig 2:**
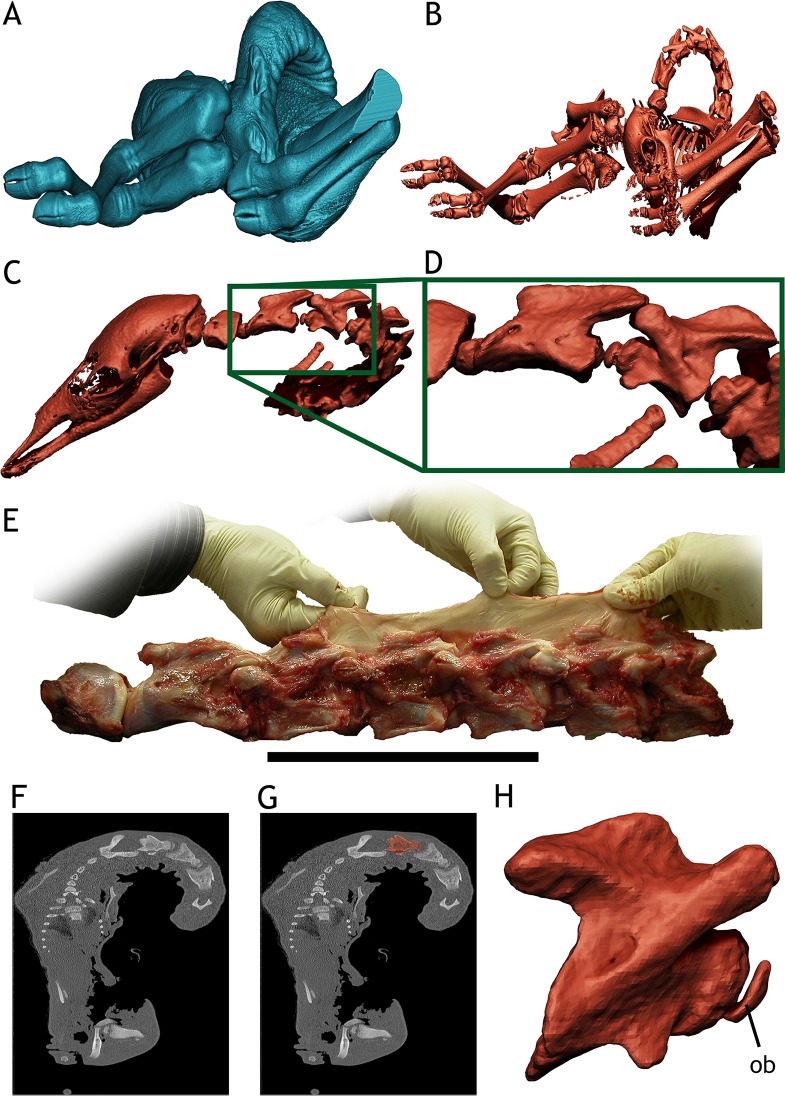
*Giraffa camelopardalis* newborn TMM M-16050. A - 3D render of the newborn giraffe body as it was CT-scanned in approximate ventral view. B - 3D render of the newborn giraffe skeleton from CT data, same view as A. C—Cervical vertebrae and skull of the newborn giraffe as it was CT-scanned, showing the intervertebral space. D—Close up on Axis-CV3 of the newborn giraffe, showing the intervertebral space and the ossification body anterior to the condyle. E—Dissected neck from a different newborn giraffe, showing the separation between vertebrae is similar to that of TMM M-16050, modified from Taylor & Wedel [12]. F—CT-scan slide of TMM M-16050. G—Highlight of CV5 on the CT-scan slide from F, showing the ossification body anterior to the vertebral centrum. H - 3D render of CV5 from CT data, showing the ossification body (ob). Scale for E = 200 mm.

Three sauropod dinosaur specimens and two giraffe specimens were studied (Fig 1). The two specimens of *Giraffa camelopardalis* represent two different ontogenetic stages. Both specimens were digitized via CT-scan. The juvenile specimen (TMM M-16050), a dead newborn from the Gladys Porter Zoo, was CT-scanned post necropsis (Fig 2), then skeletonized and donated to the University of Texas Vertebrate Paleontology collections. The scan files are deposited in the online repository MorphoSource by Duke University. The adult specimen is represented by 7 cervical vertebrae (CV) and 1 thoracic (T) vertebra deposited at the American Museum of Natural History. It was CT-scanned for an exhibition in the early 2000s (Kent Stevens, pers.comm. 2018) and has been used in previous studies regarding giraffe osteological range of motion [22]. The 3D files were loaned generously by AMNH and Kent Stevens. The actual specimen number or scanning specifications were not available.

The three specimens of *Spinophorosaurus nigerensis* also represent different ontogenetic stages (Fig 1). All specimens were collected in an area north of the Rural Community of Aderbissinat (Thirozerine Dept., Agadez Region, Republic of Niger), stratigraphically below the outcrops of the Tegama Group in the “Falaise de Tiguidit”. Holotype and paratype were found in the same level of this layer, about 15 meters laterally apart from each other on the upper unit [17].

The holotype specimen (GCP-CV-4229 and NMB-1699-R) preserves most of the skeleton, only missing most of the preorbital region of the skull, the atlas and distalmost caudal vertebrae, one coracoid, both sternal plates (if they were ossified), forelimbs, distal left ischium, both feet and left hindlimb. It is possibly a subadult specimen, as it has unfused arches and centra in most vertebrae and sutures are still visible in those vertebrae with fused centra and arches [23]. However, since neurocentral suture fusion may not be the most reliable indicative of ontogenetic state [24,25], its subadult status must be considered tentative and to be tested by histological sampling in the future. However, thorough this paper both "subadult" and "holotype" will refer to this specimen.

The paratype specimen (NMB-1698-R) preserves fewer elements than the holotype, among them six cervical vertebrae, four with remarkably well-preserved pre- and postzygapophyses. It is not a fully grown adult, but appears to be in a more advanced stage in its ontogeny than the holotype: most bones are larger than the same bones on the holotype, the neurocentral sutures are closed and not visible, but the cervical ribs and scapula and coracoid are unfused. Recent histological analyses have found it not only to be an adult, but also to have survived an osteological tumor-like pathology which caused radial fibrolamellar bone [26].

A third, likely juvenile specimen (GCP-CV-BB-15), includes an almost complete neck from the axis to the first dorsal vertebra, with one cervical vertebra (CV6) very damaged. These vertebrae share most (but not all) of the diagnostic characters of *Spinophorosaurus* [19] (under description by AP). The neurocentral sutures are open, the centra are less elongated than those on the holotype and paratype and its vertebrae are a quarter the size of the holotype vertebrae. The third specimen was found *ex-situ* in the surface, but with most elements articulated and no duplicity in vertebrae, so it can be considered a single specimen. The lithology of the matrix containing this specimen is identical to the one where the holotype and paratype were excavated, it was found near the upper layer that contained both holotype and paratype specimens and there is an absence of relief in the area. This likely means that all three specimens come from the same horizon.

## Methods

### Preparing the virtual skeletons

For the juvenile specimen of *Giraffa camelopardalis* (TMM M-16050), the CT-Scan data was first imported to ImageJ 1.49b (National Institutes of Health, USA, 2014) for artifact removal and optimizing contrast. Then, it was imported into Avizo 7.1.0 (VSG, Burlington, MA, USA, 2013) for segmentation and 3D reconstruction. The soft tissues were reconstructed as a whole mesh and exported in Wavefront OBJ format (Fig 2A). Each cervical vertebra and the first two dorsal vertebrae were reconstructed individually, slice per slice, and exported as a high-resolution Wavefront OBJ model in their original position (Fig 2C). The remaining dorsal vertebrae and ribs were reconstructed as a whole, single structure, and exported as a high resolution Wavefront OBJ. The same procedure was followed with the sacrum and pelvis, each limb and the skull. Overall we reconstructed as individual models: seven cervical vertebrae, two dorsal vertebrae, one mesh of dorsal vertebrae + ribs, one mesh with the sacrum + pelvic girdle + caudal vertebrae, two meshes of pectoral girdle + forelimbs, two meshes of hindlimbs.

The fossil *Spinophorosaurus* specimens were surface-scanned by photogrammetry, following the protocol of Mallison and Wings [27] in Photoscan 1.3 (Agisoft LLC

11 Degtyarniy per., St. Petersburg, Russia, 191144). Unlike the holotype, most cervical vertebrae of the juvenile were easily separated physically from each other during preparation, with the exception of vertebrae CV11 and CV10, which had to be digitally separated in ZBrush 4R6.(Pixologic, USA, 2013). The mesh was duplicated as two separate sub-tools. Using the mask-lasso brush, each vertebra was masked (a different one in each of the models) and then the trim-lasso brush was used to eliminate the unmasked vertebra. The missing portions (prezygapophysis medial side, anterior centrum condyle and pedicels in CV11; postzygapophyses, posterior centrum cotyle and pedicels in CV10) were digitally sculpted based upon preceding and following vertebrae. CV12 right prezygapophysis was broken, physically attached to CV11. It was digitally separated following the same procedure to separate CV11 and CV10. The articular facet was visible and needed no restoration, unlike the rest of the medial side, which was not visible, and was sculpted based upon that of DV1.

The subadult skeleton was assembled from the holotype specimen, with missing elements stemming from the paratype (humerus, scaled to have the same humerus/scapula ratio as in the paratype) and closely related sauropods (with forelimb and feet proportions close to those from *Shunosaurus* [28], *Mamenchisaurus* [3] or *Jobaria* [29]). Since many cervical vertebrae could not be separated from the preceding vertebra during preparation, the medial portion of many prezygapophyseal facets is obscured. To estimate the medial side of the prezygapophyses, the visible lateral portion was used as the posteriormost limit for the prezygapophyseal facet, with the rest of the facet reconstructed by digitally sculpting the missing medio-dorsal part of the facet (area between visible dorsal side of the facet and the ventro-medial, non-facet side of the prezygapophyses). This was done following the same protocol described above for the juvenile specimen.

### Assembling the virtual skeletons

**Fig 3:**
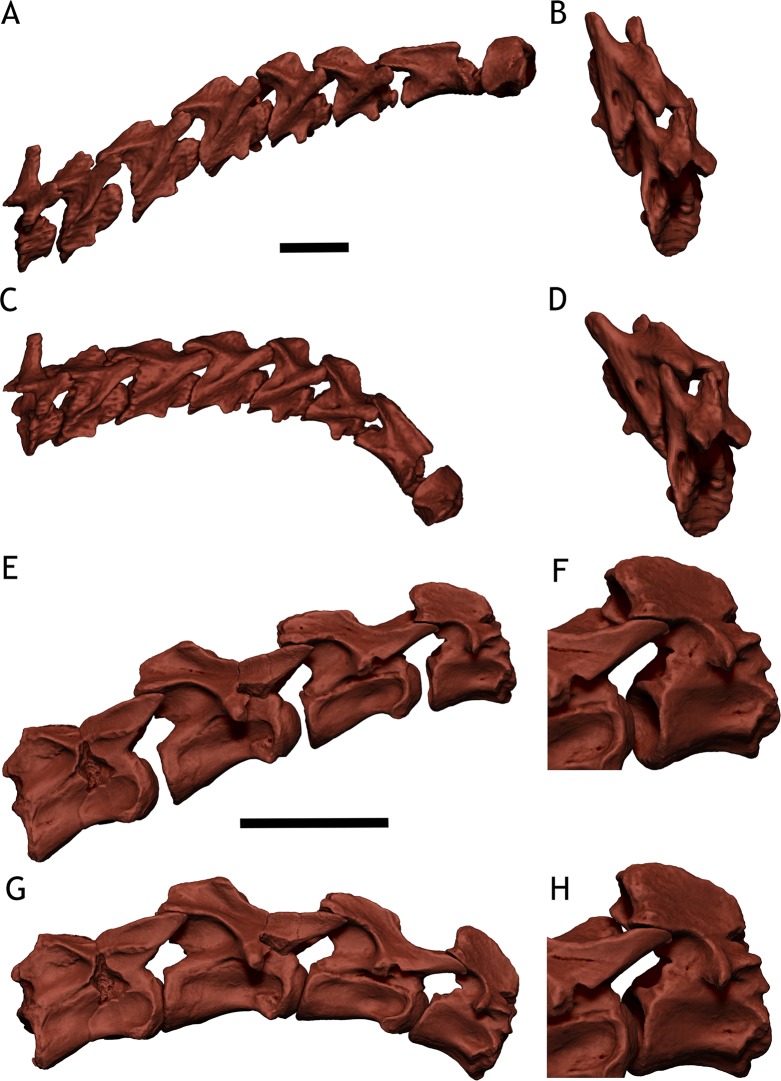
Comparing osteologically neutral pose (ONP) and neutral bone only posture (NBOP). Notice the vast difference between the osteologically induced curvature (OIC) and amount of intervertebral space rendered by ONP and those rendered by NBOP. A—Newborn *Giraffa* neck in ONP B—CV7 and CV6 in postero-lateral view in ONP, notice the full zygapophyseal overlap. C—Newborn *Giraffa* neck in NBOP. D—CV7 and CV6 in postero-lateral view in NBOP, notice how the zygapophyses are not fully overlapped, and have to be angled in order to have the centra rims parallel. E—Juvenile *Spinophorosaurus* CV5 to Axis in ONP. F—CV3 and Axis in postero-lateral view in ONP, notice the full zygapophyseal overlap. G—Juvenile *Spinophorosaurus* CV5 to Axis in NBOP. H—CV3 and Axis in postero-lateral view in NBOP, how the zygapophyses are not fully overlaped, and have to be angled in order to have the centra rims parallel. Scales for A, C, E and G = 50 mm.

Each skeleton was imported into ZBrush 4R6 bone by bone and articulated following the protocol of Mallison [14,15]. Overall, the axial skeleton was articulated in pairs, with only two elements visible at once in order to minimize preconceived notions: one remained static while the other was articulated in Osteologically Neutral Pose (ONP; Fig 3). A majority of authors have defined ONP as the maximum alignment of the zygapophyses [8,9,11,14,15,22,30]. On the other hand, in addition to the maximum overlap of the zygapophyses, some authors implemented that anterior and posterior facets of vertebral centra had to be parallel in their definitions of ONP [10,31,32]. While intervertebral soft tissue might vary in relative size to the vertebral centra and it may change the geometry of the axial skeleton, the zygapophyseal capsules are not thicker than a flat sheet with the same outline as the bony facet, not affecting the geometry of the axial skeleton as much [12].

Our usage of the term ONP refers only to the zygapophyseal joints, given i) the discrepancy between zygapophyseal and zygapophyseal + centra alignments, ii) the more widespread use of the maximum zygapophyseal overlap criterion alone and, iii) the fact that intervertebral soft tissue thickness is more variable than zygapophyseal capsule thickness. In this work, ONP is the full articulation of the zygapophyseal facets, with complete overlap of the facet outlines in all three anatomical planes (antero-posterior, lateral-medial, dorso-ventral). Neutral Bone Only Posture (NBOP *sensu* Paul [21]) was coined for accounting not only for the best possible articulation of pre- and postzygapophyseal facets, but also to have the rims of the vertebral centra parallel (Fig 3; see discussion).

If both skeletal assemblages (that performed from anterior to posterior and vice-versa) had the same Osteologically Induced Curvature (OIC, sensu Stevens [22]; “the curvature of a vertebral column in ONP, as distinguished from curvature induced by joint deflection”) the virtual mount was considered positive and ready for ROM analysis.

The appendicular skeleton was articulated by pairs following Reiss and Mallison [33], in ONP (long axes of two bones articulating with each other were placed approximately parallel to each other in lateral view). Since the axial skeleton fossils of the juvenile *Spinophorosaurus* are small and stable, it was also articulated in pairs manually, the results compared with those obtained in the virtual workspace.

All measurements relative to neck and head heights and angles were taken relative to the ground. The ground was set as 0° (horizontal) for measuring angles and as 0 meters for measuring heights. In the case of the adult giraffe, for which we do not have a complete skeleton, we used a 3D full body flesh model of a giraffe, scaled to have the same neck length. In the case of the juvenile *Spinophorosaurus*, for which we have a less complete skeleton, we used an isometrically scaled-down post-cervical virtual skeleton of the adult (Fig 1), assuming limbs grew isometrically [34]. This placeholder skeleton was scaled to the same DV1 size. To measure the angles between two individual vertebrae, we used the angulation between the same landmark of two consecutive vertebrae (see "Measuring inter-vertebral range of motion" below for a more detailed explanation).

### Measuring inter-vertebral space

Intervertebral space was measured in two different ways with the virtual bones in ONP in ZBrush 4R6 (Pixologic, USA, 2013). First, a direct measurement of the gap separating two vertebrae was measured, as the maximum distance between the deepest region of the cotyle of the anterior vertebra and the anteriormost part on the condyle of the posterior vertebra. An indirect measurement was also made by measuring the complete length of the segment (as the distance between the deepest region of the cotyle of the posterior vertebra and the anteriormost part on the condyle of the anterior vertebra) and the individual length of both vertebrae in the segment (also deepest cotyle to anteriormost condyle, "functional length", see ninth figure in Taylor and Wedel [12]). The difference between the complete length of the segment and the addition of both vertebrae lengths would correspond to the intervertebral space:

CompleteSegmentLength‐(AnteriorVert.Length+PosteriorVert.Length)=Intervertebralspace

The difference between the direct measurement and the indirect measurement turned out to be minimal. The percentage of the intervertebral space is the percentage of the length of the corresponding measured segment (Table 1).

**Table 1:** Amount of neck intervertebral space. Percentages measure the amount of the segment length corresponding to intervertebral space.

Specimen	Greatest	Lowest	Average (whole neck)
Newborn *Giraffa* (articulated)	52%	11.6%	27.27%
Newborn *Giraffa* (ONP)	45%	12.3%	27.03%
Adult *Giraffa* (ONP)	6%	4%	4.8%
Juvenile *Spinophorosaurus* (ONP)	16%	11.1%	13.5%
Adult *Spinophorosaurus* (ONP)	6%	3%	4.5%

### Measuring inter-vertebral range of motion

The osteological range of motion (ROM) was calculated by deflecting each vertebra one pair at a time, with the posteriormost one remaining static while the anteriormost one was deflected. There has been some discrepancies on how much pre- and postzygapophyseal articular facets may deflect before disarticulation. Early on, the zygapophyseal safety factor, the minimal overlap of the facets before there is too much strain on the zygapophyseal articular capsules (*sensu* Stevens [22]), was set to be at around 50% of overlap [8]. However, observations on extant crocodiles [15] or birds [35] show that living extant animals can attain postures with little overlap between their zygapophyseal facets.

For this study, we decided to follow the protocols of Mallison [15], in which all vertebrae, in the absence of osteological stops, were deflected until only a minimum overlap of the facets was retained. Beyond that point, they were considered disarticulated. That way, accounting a larger facet *in vivo* with a fresh zygapophyseal capsule [12], the range of motion is underestimated rather than overestimated, in accordance with what happens in present day archosaurs. In lateral flexion, zygapophyseal overlap was retained in three different ways: (i) maintaining one zygapophyseal pair in full articulation and deflecting the other pair anteriorly, (ii) maintaining one zygapophyseal pair in full articulation and deflecting the other pair posteriorly, and (iii) deflecting one zygapophyseal pair posteriorly and the other pair anteriorly, which would be the maximum range of motion before disarticulation. Both (i) and (ii) rendered similar range of motion at all intervertebral joints, so all figures depicting lateral range of motion of the neck depict (i) and (iii).

To measure the angles between two individual vertebrae, we used the angulation between the same landmark of two consecutive vertebrae: the posteriormost and ventralmost point of the vertebral centrum in lateral view for dorsoventral ROM, and the right prezygapophyses in dorsal view for lateral ROM. These were later contrasted against a different method: drawing straight lines parallel to the main axis of the vertebral centrum in lateral (dorsoventral ROM) or the neural spines in dorsal view (lateral ROM) and measuring the angle they make, which was the same as the one measured between landmarks in all cases.

As with the OIC, the juvenile *Spinophorosaurus* specimen range of motion was manually measured with the original fossils and the results compared favorably with those obtained in the virtual workspace.

## Results

### *Giraffa camelopardalis* newborn

Since the scanned skeleton of the newborn giraffe comes from an articulated dead specimen (Fig 2A and 2B), we made the following observations of the scan before digitally separating the bones: (i) the intervertebral space between cervical vertebrae and caudal vertebrae pairs (Fig 2B–2D) is much greater than the one between dorsal and sacral pairs. (ii) There is an ossification body in the anterior centrum of most cervical vertebrae. This body may be slightly separated from the actual condyle but is usually connected by an ossified constriction in most vertebrae (Fig 2F–2H). (iii) The mineralized bone matrix of some cervical vertebrae is asymmetrical, that is, one of the sides (left or right) is more ossified than the other. (iv) The pose of the neck, flexed ventrally and laterally to the right with some torsion, makes the head reach the torso and required the left pre- and postzygapophyseal facets to be completely disarticulated from Axis-CV3 to CV6-CV7 joints (Fig 2C). The ribs of the right side are misaligned from their proximal diaphyses, where they were apparently cut when necropsy was performed. The ossified epiphyses and diaphyses of long bones are well separated and unfused.

We measured the intervertebral space on the cervical and anterior dorsal vertebrae on the virtual skeleton as it was scanned in order to have some measurements without any manipulation of the bones. The intervertebral space accounted for about 27.27% the length of the Axis-T2 segment, with the amount of intervertebral space ranging between 18.2 mm. and 8.7 mm (Table 1).

After articulating the neck in osteologically neutral pose (ONP; Fig 3A), the intervertebral space was present in all vertebrae pairs, albeit with some differences in the exact intervertebral space amount from the non-manipulated pose (Table 1). The intervertebral space accounted for 27.03% of the length of the Axis-T2 segment in ONP, ranging between 17.9 mm. and 8.9 mm (Table 1).

**Fig 4:**
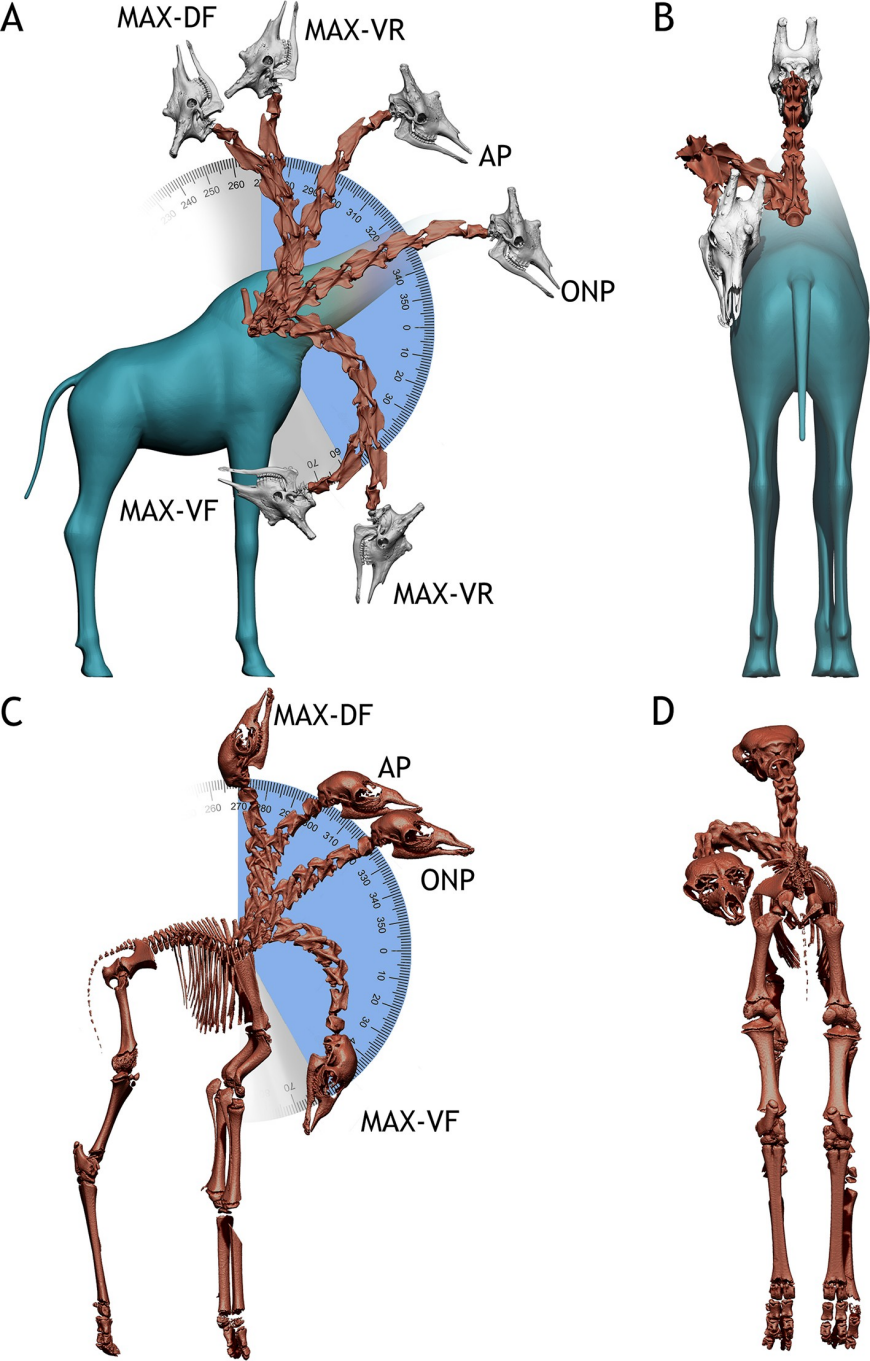
*Giraffa camelopardalis* neck postures. A—Several postures within the dorso-ventral osteological range of motion of the adult *Giraffa* in lateral view. Blue indicates the arc described between maximum and minimum head heights. Grey indicates the arc described between maximum dorsiflexion and ventriflexion. B—Adult *Giraffa* in posterior view, with the neck folded against the body, achieved with full lateral flexion (III) of T1-CV7, CV7-CV6, and CV6-CV5 joints. C—Several postures within the dorso-ventral osteological range of motion of the newborn *Giraffa* in lateral view. Blue indicates the arc described between maximum and minimum head heights. Grey indicates the arc described between maximum dorsiflexion and ventriflexion. D—Newborn *Giraffa* in posterior view, with the neck folded against the body, achieved with full lateral flexion (III) of T1-CV7, CV7-CV6, and CV6-CV5. Max-DF = maximum dorsiflexion. Max-VR = maximum vertical reach. AP = alert posture. ONP = osteologically neutral pose. Max-VR = maximum ventral reach. Max-VF = maximum ventriflexion. Red bones = main specimen. White bones = extrapolated elements (see methods). Blue = flesh model. Angles in degrees.

The osteologically induced curvature (OIC) of the newborn giraffe dorsal vertebrae series shows a slightly bowed spine, with an angle of only 5° between the first sacral centrum and the first dorsal centrum (T1). The neck, on the other hand, has a steeper angle (Fig 4C), forming a 39° angle between T1 and the atlas. This is caused by greatly wedge-shaped T2, T1 and CV7 vertebrae, which account for most of the verticalization of the neck (the angle between the centra of T2-CV6 is already 40°). From CV5 to Atlas, the OIC is slightly ventriflexed, with a -1° angle between CV5 and the atlas. The muzzle of the newborn giraffe is at a height of 1.12 meters from the ground in ONP (with T1 at 0.91 meters and the shoulder joint at 0.72 meters).

**Fig 5:**
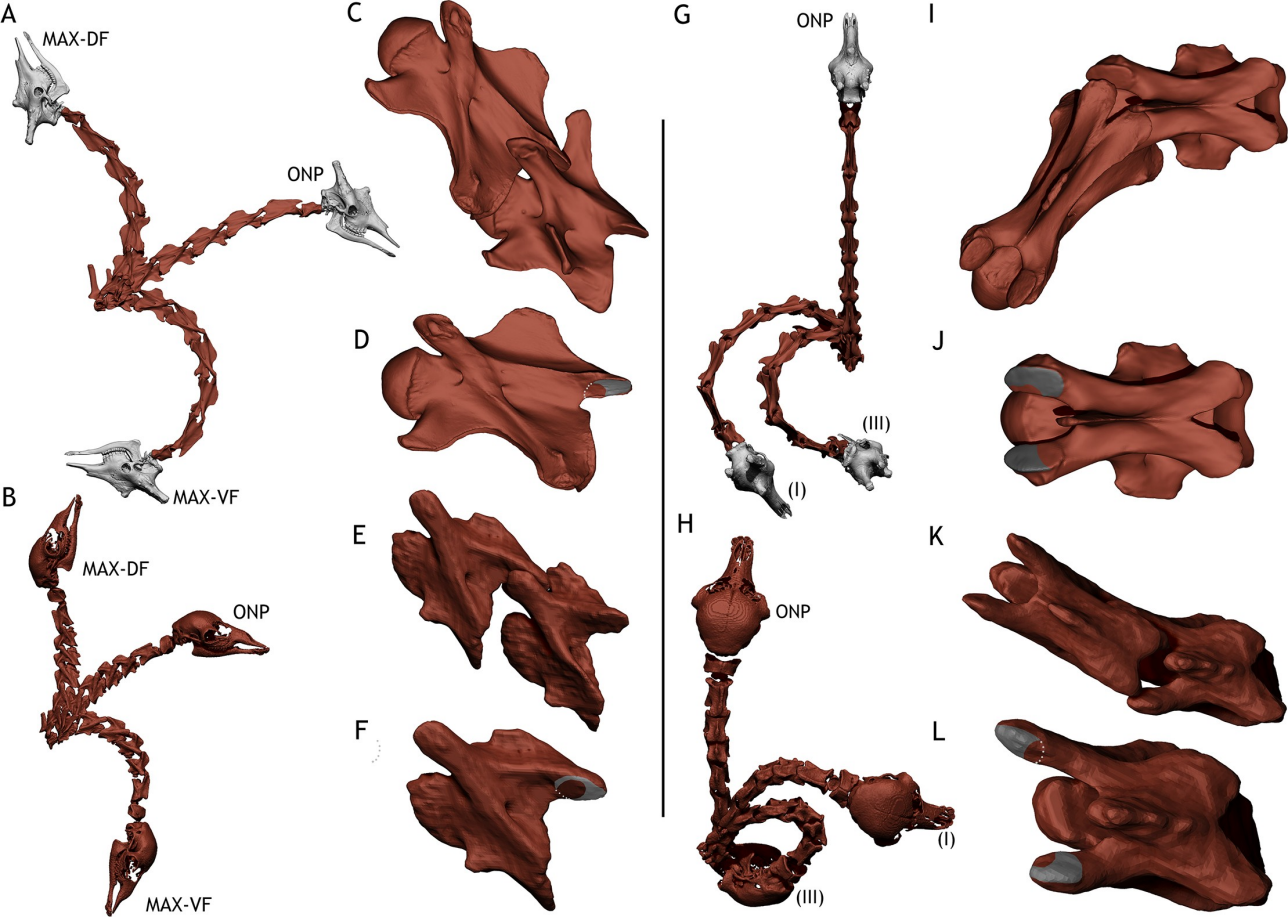
*Giraffa camelopardalis* ostelogical neck range of motion. A—Dorso-ventral osteological range of motion of the adult *Giraffa* in lateral view. B—Dorso-ventral osteological range of motion of the newborn *Giraffa* in lateral view. C—CV7-CV6 joint of the adult *Giraffa* in maximum dorsiflexion (Max-DF) in lateral view. D—CV6 of the adult in lateral view *Giraffa* showing how much of the postzygapophysis overlaps with the prezygapophysis of CV7 in maximum dorsiflexion (in red, zygapophyseal overlap, in gray non overlapped area of the facet). E—CV7-CV6 joint of the newborn *Giraffa* in maximum dorsiflexion (Max-DF) in lateral view. F—CV6 of the newborn *Giraffa* in lateral view showing how much of the postzygapophysis overlaps with the prezygapophysis of CV7 in maximum dorsiflexion (in red, zygapophyseal overlap, in gray non overlapped area of the facet). G—Lateral osteological range of motion of the adult *Giraffa* in dorsal view. H—Lateral osteological range of motion of the newborn *Giraffa* in dorsal view. I—CV7-CV6 joint of the adult *Giraffa* in maximum lateral flexion (III) in dorsal view. J—CV7 of the adult *Giraffa* in dorsal view showing how much of the prezygapophysis overlaps with the postzygapophysis of CV6 in maximum dorsiflexion (in red, zygapophyseal overlap, in gray non overlapped area of the facet). K- CV7-CV6 joint of the newborn *Giraffa* in maximum lateral flexion (III) in dorsal view. L—CV6 of the newborn *Giraffa* in dorsal view showing how much of the prezygapophysis overlaps with the postzygapophysis of CV6 in maximum dorsiflexion (in red, zygapophyseal overlap, in gray non overlapped area of the facet). Red bones = main specimen. White bones = extrapolated elements (see methods). Max-DF = maximum dorsiflexion. ONP = osteologically neutral pose. Max-VF = maximum ventriflexion. I = lateral flexion attained when maintaining one zygapophyseal pair in full articulation and deflecting the other pair anteriorly. III = maximum lateral flexion.

The osteological range of motion (ROM) of the neck in dorsiflexion allows the neck to be placed vertical and a little beyond, with the atlas forming a 91° angle with T1 at full dorsiflexion. This dorsiflexion allows the muzzle to reach a maximum height of 1.47 meters. Maximum ventriflexion makes the atlas form an angle of -46° with T1. This allows placing the muzzle at 0.49 meters from the ground (Fig 4C). The range of motion in lateral flexion allows the head to touch the body without disarticulating, with the head colliding with the body at full range of motion. (Fig 4D) However, it is necessary to deflect both zygapophyseal articulations to allow the head to reach the body (Fig 5H–5L). When only one pair of pre- and postzygapophyseal facets are deflected before disarticulation (ii, see methods), the arc described by the neck is of around 90°, but it does not allow pointing the muzzle posteriorly (that is, 180° from ONP, Fig 5H).

### *Giraffa camelopardalis* adult

The intervertebral space is significantly smaller in the adult specimen than in the newborn, at least on the 7 cervical vertebrae, in ONP. The condyles fit deeply in the cotyles. The intervertebral space accounted for about 4.8% the length of the Axis-T1 segment (Table 1).

The OIC of the adult giraffe neck has a steep angle (Fig 4A), forming a 26° angle between T1 and the atlas (Fig 4A, Fig 5A). Most of this deflection occurs at the T1-CV7 and CV7-CV6 joints, since the centra of T1 and CV7 are wedge-shaped, and therefore dorsally deflect the vertebrae anterior to them in ONP. The remaining cervical vertebrae centra are not wedged, and therefore articulate almost straight, although very slightly dorsally deflected (about 2° between CV6 and the atlas).

The osteological ROM of the adult giraffe neck is greater in all respects compared with that of the newborn (Fig 4). In maximum dorsiflexion, the atlas can deflect up to 103° from T1 vertebra before disarticulation. In maximum ventriflexion, the atlas can deflect up to -73° from T1 before disarticulation. The muzzle can therefore be situated at 90° respective to the ground before disarticulation occurs, at 4.1 meters. It is, however, impossible for the muzzle to reach the ground by ventriflexion of the neck alone, reaching a minimum height of 0.45 meters. In lateral flexion, the neck can describe a curve of more than 180°, with the head reaching the body well before disarticulating any of the cervical vertebrae joints (Figs 4B and 5G).

### *Spinophorosaurus nigerensis* juvenile

**Fig 6:**
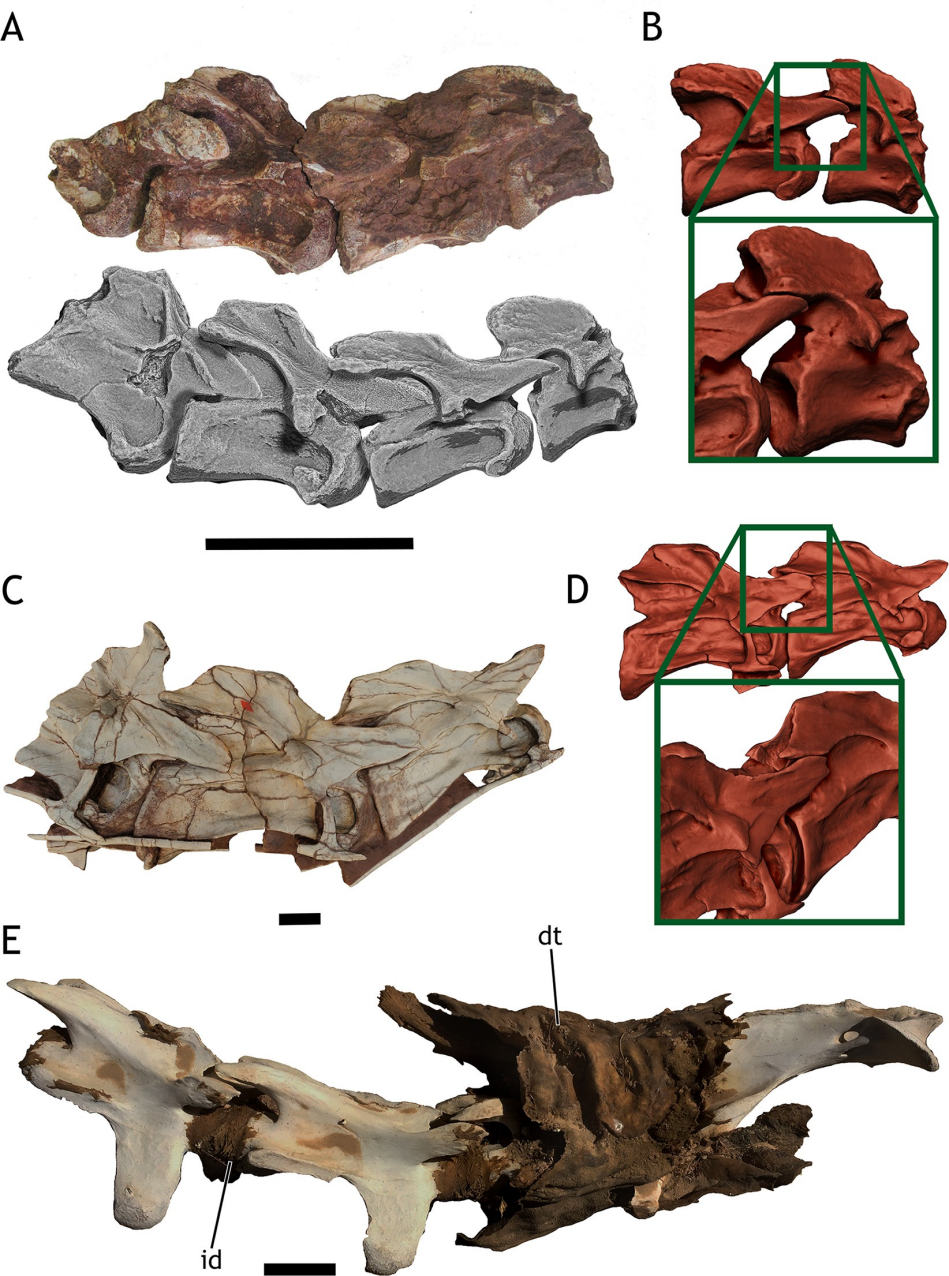
*Spinophorosaurus nigerensis* neck fossils. A—Articulated anterior segment of the neck (CV5-Axis) from the juvenile *Spinophorosaurus* before preparation in lateral view (picture and 3D model articulated mimicking the field pose. Notice the condyles barely fit inside the cotyles). B—CV3-Axis joint from the juvenile *Spinophorosaurus* in ONP (zygapophyses in full overlap) in lateral view. Notice the intervertebral space. C—Articulated segment of the neck (CV7-CV5) from the subadult holotype *Spinophorosaurus* (Notice the condyles are more deeply nested inside the cotyles than in the juvenile) in lateral view. D—CV6-CV5 joint from the subadult *Spinophorosaurus* in ONP (zygapophyses in full overlap) in lateral view. Notice there is barely any intervertebral space, and the condyles are nested within cotyles. E—Articulated anterior segment of the neck (CV5-Axis) from a subadult specimen of *Camelus dromedarius* in lateral view photographed in the Sahara desert, showing the presence of intervertebral space (still filled with the intervertebral disc) remains despite desiccation in an arid environment. Scale bars = 50 mm. dt = desiccated tissue. id = intervertebral disc.

The best-preserved vertebrae of the juvenile *Spinophorosaurus* are the axis, CV3, CV4, CV12 and DV1. The remaining vertebrae either lack pre-postzygapophyses or are too distorted for virtual or manual articulation to be completely reliable. However, the fossil was found in articulation and photographed before preparation (Fig 6A), so a non-manipulated intervertebral space can be roughly assessed and compared with that obtained in the virtual mount.

The OIC reveals a significant amount of intervertebral space among the best-preserved vertebrae (Fig 3E and 3F). The amount of intervertebral space in the Axis-CV5 segment is 12.4% of its length, with a maximum of 17.96 mm and a minimum of 11.28 mm. Between the CV7-CV8 centra there is 18.77 mm of intervertebral space, which accounts for 12.6% the length of that segment. Between the CV12-D1 centra there is 18.87 mm of intervertebral space, accounting for 15.86% the length of that segment. Pictures of the fossils before preparation all show some intervertebral space, since the condyles barely fit into the cotyles (Fig 6A).

**Fig 7:**
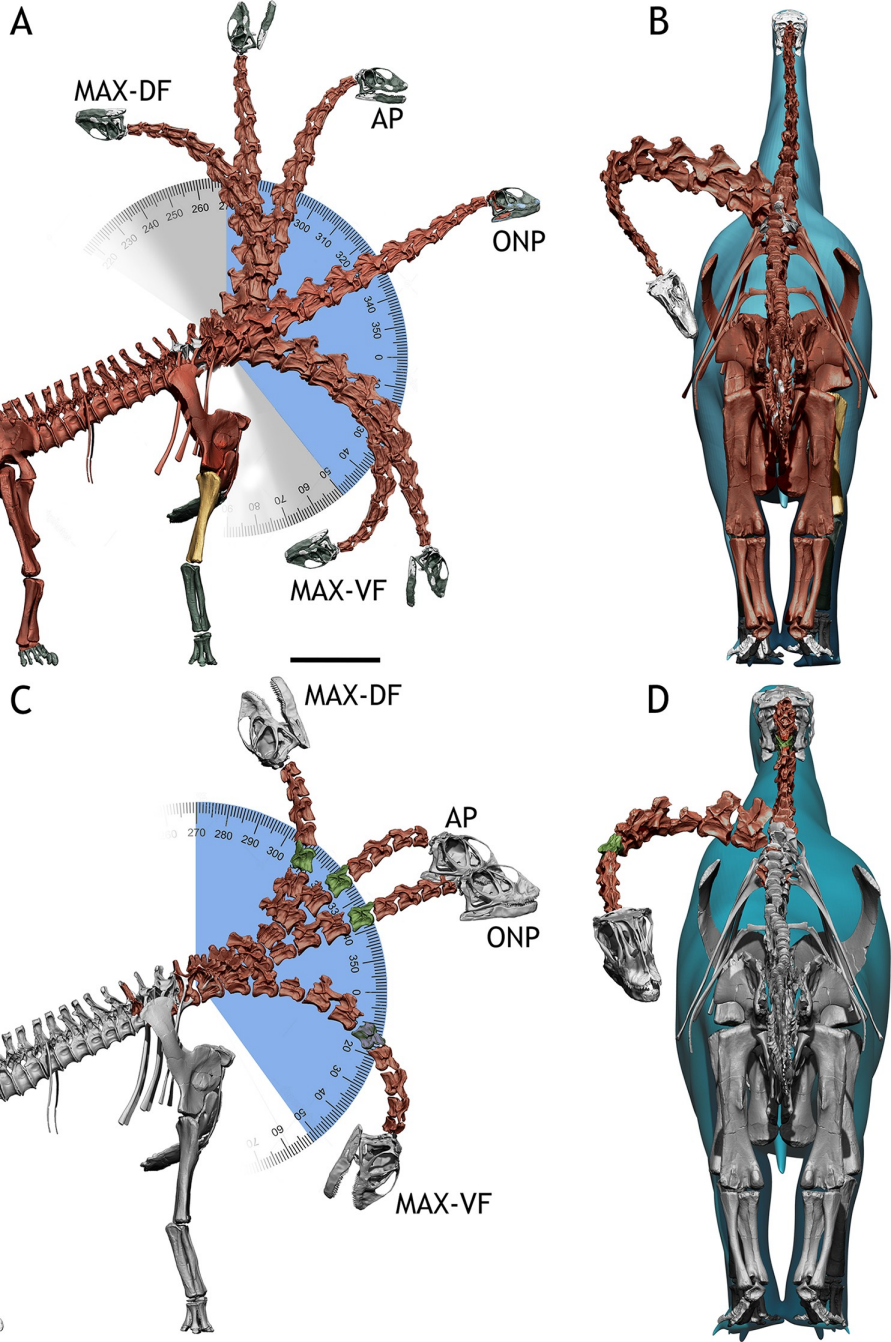
*Spinophorosaurus nigerensis* neck postures. A—Several postures within the dorso-ventral osteological range of motion of the subadult *Spinophorosaurus*. Blue indicates the arc described between maximum and minimum head heights. Grey indicates the arc described between maximum dorsiflexion and ventriflexion. B—Subadult *Spinophorosaurus* in posterior view, with the neck folded against the body. C—Several postures within the dorso-ventral osteological range of motion of the juvenile *Spinophorosaurus*. Blue indicates the arc described between maximum and minimum head heights. Grey indicates the arc described between maximum dorsiflexion and ventriflexion. D—Juvenile *Spinophorosaurus* in posterior view, with the neck folded against the body. Max-DF = maximum dorsiflexion. AP = alert posture. ONP = osteologically neutral pose. Max-VF = maximum ventriflexion. Red bones = main specimen. White bones = hypothetical scaled elements (see methods). Green Bones = vertebrae modeled after the closest complete element. Yellow Bones = bones modeled after different sized specimens (see methods). Blue = flesh model. Angles in degrees.

**Fig 8:**
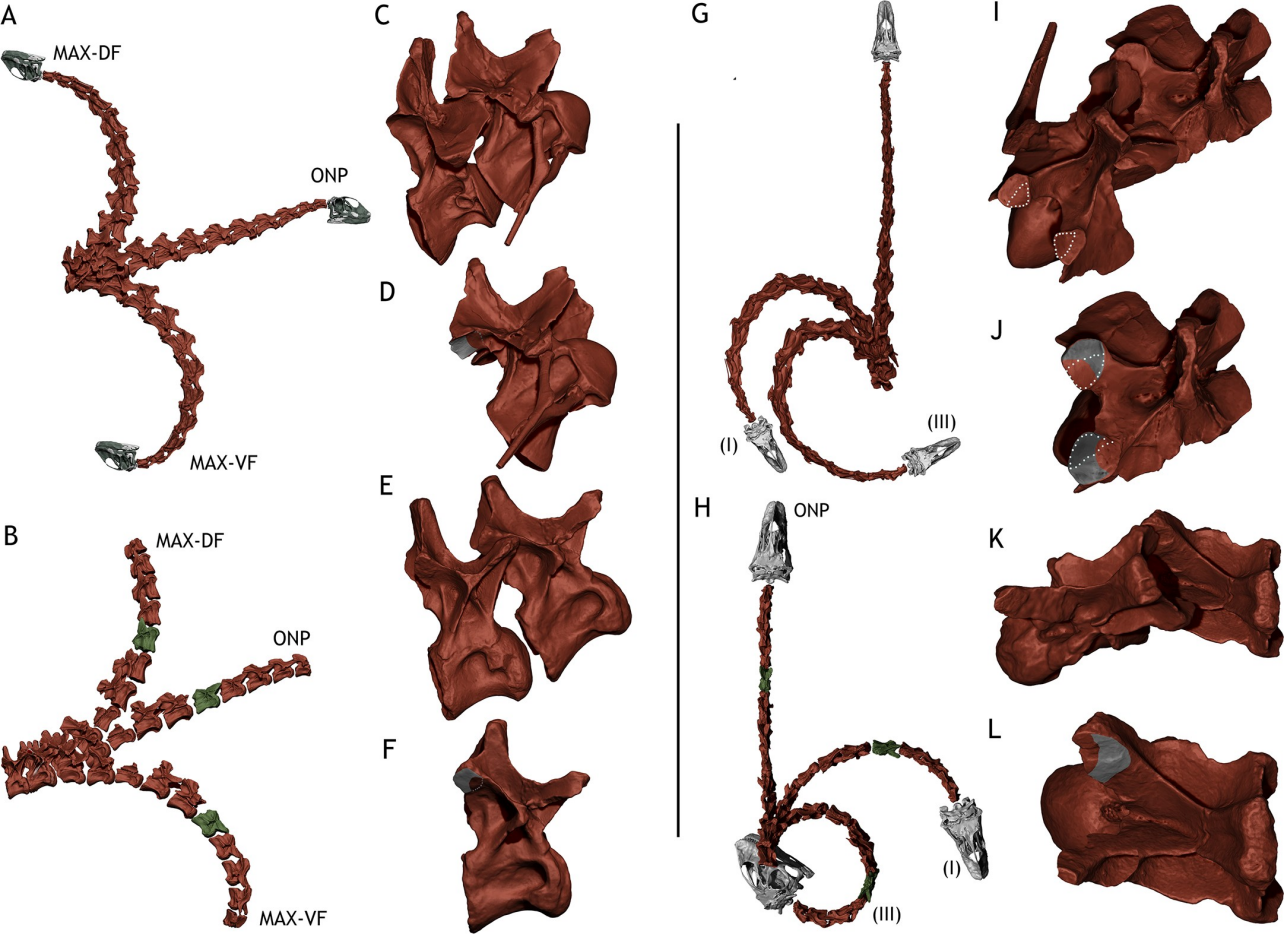
*Spinophorosaurus nigerensis* neck ostelogical range of motion. A—Dorso-ventral osteological range of motion of the subadult *Spinophorosaurus* in lateral view. B—Dorso-ventral osteological range of motion of the juvenile *Spinophorosaurus* in lateral view. C—DV1-CV12 joint of the subadult *Spinophorosaurus* in maximum dorsiflexion (Max-DF) in lateral view. D—CV12 of the subadult *Spinophorosaurus* in lateral view showing how much of the postzygapophysis overlaps with the prezygapophysis of DV1 in maximum dorsiflexion (in red, zygapophyseal overlap, in gray non overlapped area of the facet). E—DV1-CV12 joint of the juvenile *Spinophorosaurus* in maximum dorsiflexion (Max-DF) in lateral view. F—CV12 of the juvenile *Spinophorosaurus* in lateral view showing how much of the postzygapophysis overlaps with the prezygapophysis of DV1 in maximum dorsiflexion (in red, zygapophyseal overlap, in gray non overlapped area of the facet). G—Lateral osteological range of motion of the subadult *Spinophorosaurus* in dorsal view. H—Lateral osteological range of motion of the juvenile *Spinophorosaurus* in dorsal view. I -D1-CV12 joint of the subadult *Spinophorosaurus* in maximum lateral flexion (III) in dorsal view (dots indicate estimated region of the prezygapophyseal facet). J—DV1 of the subadult *Spinophorosaurus* in dorsal view showing how much of the prezygapophysis overlaps with the postzygapophysis of CV12 in maximum dorsiflexion (in red, zygapophyseal overlap, in gray non overlapped area of the facet, dots indicate estimated region of the prezygapophyseal facet). K- DV1-CV12 joint of the juvenile *Spinophorosaurus* in maximum lateral flexion (III) in dorsal view. L—DV1 of the juvenile *Spinophorosaurus* in dorsal view showing how much of the prezygapophysis overlaps with the postzygapophysis of CV12 in maximum dorsiflexion (in red, zygapophyseal overlap, in gray non overlapped area of the facet). Max-DF = maximum dorsiflexion. ONP = osteologically neutral pose. Max-VF = maximum ventriflexion. I = lateral flexion attained when maintaining one zygapophyseal pair in full articulation and deflecting the other pair anteriorly. III = maximum lateral flexion. Red bones = main specimen. White bones = extrapolated elements (see methods). Green Bones = vertebrae modeled after the closest complete element.

The osteological range of motion of the juvenile *Spinophorosaurus* can only be accurately estimated in the axis-CV5 segment and the CV7-CV8 and DV1-CV12 joints, since those preserve pre- and postzygapophyses. In order to obtain an approximation of the complete neck range of motion, missing joints have been estimated to have as deflection the arithmetic mean of the closest preceding and following joint angles (Table 2). In full dorsiflexion, the curve described by the neck allows the head to be situated vertically, at 90° (at an angle of 79° with DV1). This dorsiflexion allows the muzzle to reach a maximum height of 1.83 meters. Maximum ventriflexion makes the axis form an angle of -53° with DV1. This allows placing the muzzle at 0.35 m from the ground (Fig 7C). The range of motion in lateral flexion allows the head to touch the body without disarticulating (Fig 8H, 8K and 8L), with the head actually colliding with the body before reaching the maximum lateral flexion (Fig 7D). When only one pair of pre- and postzygapophyseal facets are deflected before disarticulation, the arc described by the neck is of around 80°, so it does not allow pointing the muzzle posteriorly (Fig 8H).

**Table 2:** Osteological dorso-ventral neck range of motion. Angles are measured in degrees, and represent the range between maximum dorsiflexion and maximum ventriflexion. An asterisk (*) indicates that joint could not be measured and therefore was estimated based on the arithmetic mean of ROM at preceding and following joint. N = newborn; A = adult; J = juvenile. CV = Cervical Vertebra. T = Thoracic Vertebra (for giraffe only). DV = Dorsal Vertebra (for *Spinophorosaurus* only).

Joint	N. *Giraffa*	A. *Giraffa*	J. *Spinophorosaurus*	A. *Spinophorosaurus*
CV2-CV3	31	34	25	35
CV3-CV4	24	48	15	28
CV4-CV5	38	46	17	25
CV5-CV6	32	39	15	21
CV6-CV7	40	44	13*	22
CV7-CV8/T1	34	30	12	23
CV8-CV9	-	-	13*	18
CV9-CV10	-	-	14*	21
CV10-CV11	-	-	15	28
CV11-CV12	-	-	16	27
CV12-DV1	-	-	7	17

### *Spinophorosaurus nigerensis* subadult

In the holotype of *Spinophorosaurus*, the intervertebral space in ONP is significantly lower than in the juvenile specimen. The condyles fit deeply in the cotyles, and the intervertebral space ranges from 6–2.5% in the joints it could be accurately measured, in the absence of CT-scan (Table 1). The amount of intervertebral space for the whole neck length is around 4.5% of its total length. Pictures of cervical vertebrae still in articulation show the condyles are deeply nested in the cotyles (Fig 6C). As with the juvenile specimen, the fossil was found in opisthotonic posture [17], so the amount of intervertebral space might have varied. When articulating the complete axial skeleton of the holotype of *Spinophorosaurus nigerensis*, the presacral and caudal sections of the vertebral column deflect 20°. This, in addition with the slightly wedged dorsal vertebrae and the articulation of the cervical vertebrae in ONP, makes the axis deflect 31° from the last sacral vertebra (Fig 7A, ONP).

The osteological ROM of the subadult *Spinophorosaurus* can be better estimated in the antero-posterior axis in all joints, since the medial portions of the prezygapophyses are obscured in most vertebrae, as they could not be separated from the preceding vertebra during preparation. The lateral range of motion is more dubious, so the only considered postures were those in which the overlapping portion of the prezygapophyses was not extrapolated, and was visible in the actual fossil. Despite those caveats, the postures in Figs 7 and 8 could be attained without disarticulating the visible portions of the prezygapophyseal facets.

In full dorsiflexion, the curve described by the neck allows the head to be situated beyond vertical, at around 120° from DV1. At maximum verticality (90°) the muzzle can reach a maximum height of 7.16 meters from the ground. Maximum ventriflexion makes the skull form an angle of -70° with DV1. This allows placing the muzzle at 0.72 m from the ground (Fig 7A). The range of motion in lateral flexion allows the head to touch the body without disarticulating (Fig 8A), with the head actually colliding with the body before reaching the maximum lateral flexion (Fig 7B). When only one pair of pre- and postzygapophyseal facets are deflected before disarticulation, the arc described by the neck is of around 100°, allowing to point the muzzle toward posterior (Fig 8H).

## Discussion

### Intervertebral space estimation

The amount of soft tissue in archosaur joints has been shown to potentially affect the amount of motion in a given joint [12,36,37]. Therefore, estimating the amount of soft tissue in joints is an important step in assessing the motion capabilities of extinct vertebrates. Unfortunately, although some long bone epiphyses show a different texture in juveniles in which the amount of articular soft tissue is greater than in the adults [37], there are no known identifiable osteological correlates which can help estimate the exact amount of separation between adjacent bones yet. The same is true for estimating intervertebral space, since differences on the bone texture of the cotyles and condyles of opisthocoelus vertebrae with different amounts of intervertebral space are indistinguishable [12].

Our approach of estimating intervertebral space with the osteologically neutral pose in the newborn giraffe TMM M-16050 showed two things: (i) that in the newborn specimen, only the ONP rendered an intervertebral space which came close to the intervertebral space observed in the articulated CT-Scan of the specimen and, (ii) neutral bone only posture (NBOP), in which the rims of the vertebral centra are situated parallel [21], greatly underestimates the amount of intervertebral space as observed in the articulated CT-Scan of the specimen (Fig 2C vs. Fig 3C). Also, the amount of intervertebral space measured in this newborn, both in the scan of the corpse and the virtual articulated model in ONP, is very similar to the baby giraffe physically dissected and skeletonized by Taylor and Wedel [12] (24% intervertebral space in their specimen versus around 27% in TMM M-16050).

Different criteria have been applied in the definition of ONP for vertebrae since the concept was coined. The most widespread criterion is the maximum overlap between pre- and postzygapophyseal facets [8,9,11,15,22,30]. Some authors introduced as a criterion that the centra must also be in articulation [10,32,38], but since the original definition of the concept did not include the centra in its criterion [8], a new term, NBOP, has been recently proposed [21]. Given that ONP and NBOP can render very different postures for the same neck (Fig 3) we choose to consider them different criteria for assessing osteologically induced curvatures (OIC).

The usefulness of OIC (obtained by either ONP or NBOP) as something beyond establishing a standard for comparing different axial skeletons has been called into question [11,15,30]. Our findings, however, show that ONP of the neck TMM M-16050 rendered an OIC in which the amount of intervertebral space is very similar to the one in the CT-Scan of that very specimen (27.03% in ONP vs. 27.27% in the CT-Scan of the articulated specimen). NBOP, however, rendered almost no intervertebral space (Fig 3).

In the fossil specimens of *Spinophorosaurus*, ONP also has been able to predict the presence of intervertebral space in the articulated fossils: the juvenile specimen GCP-CV-BB-15 showed intervertebral space before being disarticulated for preparation, with the condyles barely around the outer rim of the cotyles (Fig 6A). This is similar to what can be seen in dromedary camel radiographs [12] and skeletonizing corpses found in the Sahara desert (Fig 6E). ONP yielded 12–16% of the complete neck length as intervertebral space in the juvenile specimen, which is a relatively large amount. The articulated fossil of the holotype specimen of *Spinophorosaurus* showed the condyles nested inside the cotyles in all vertebrae (Fig 6C). ONP showed that, although there was up to 6% intervertebral space, the condyles are still nested inside the cotyles (Fig 6D).

However, the opisthotonic posture does not represent necessarily a habitual posture and is thought to be reached either by peri-mortem contractions [39] or by post-mortem events [40]. This, together with the desiccation of vertebrate remains during decay [41] makes the intervertebral space observed in fossils likely smaller than *in vivo*. However, both ONP and the articulated opisthotonic posed, non-manipulated fossils reveal the juvenile *Spinophorosaurus* had greater inververtebral space than the adult specimen. We can claim ONP is a good criterion to roughly estimate the relative amount of intervertebral space. All in all, ONP is a close proxy to the cartilaginous neutral pose (CNP) proposed by Taylor [20], since it can roughly predict the amount of intervertebral space.

### Intervertebral space and range of motion ontogenetic changes

As both extant giraffes and *Spinophorosaurus* have ontogenetic differences between the amount of intervertebral space and the amount range of motion in cervical vertebrae, heterochrony may explain both. Peramorphosis processes have been linked to the elongation of the neck in giraffes, particularly hypermorphosis [42]. Both acceleration and hypermorphosis have been proposed as an explanation for overall size increases in many dinosaurs as well as many of their positive allometries [43]. Hypermorphosis has been determined also as the main cause for cervical vertebrae elongation in prolacertiform reptiles [44]. The amount of intervertebral space and the ossification body observed anterior to the vertebral body condyles of the newborn giraffe (Fig 2F–2H) appears to be part of the process of hypermorphosis for vertebral elongation in this taxon. Moreover, it would be possible that dromedary camels, which retain large intervertebral spaces in adulthood [12], might have them due a pedomorphic process.

Regarding *Spinophorosaurus*, the small sample known of this taxon is a caveat when testing this hypothesis, as most ontogenetic stages are not known. However, the elongation index in the subadult *Spinophorosaurus* is slightly larger than in other non-Neosauropoda Eusauropoda [17] (with the exception of Mamenchisauridae), and it is greater than in the juvenile specimen as well. Also, the biggest difference on the sizes of the individual bones between the holotype and the paratype are found among the cervical vertebrae (while the cervical vertebrae in the paratype are larger, the ilium and preserved caudal vertebrae of the paratype are about the same size as in the holotype). This suggests hypermorphosis as the most likely explanation, although more specimens in different ontogenetic stages would be needed to further test the hypothesis.

The size and shape of prezygapophyseal facets, which also determine the amount of range of motion in the neck, does not fit into the heterochronic hypothesis in both giraffes and *Spinophorosaurus*. The more elongated a prezygapophyseal facet, the more deflection can be attained before there is no pre-postzygapophyseal overlap. Given this, sauropods with more elongated prezygapophyseal facets have been shown to have greater ranges of motion per joint than those with shorter or wider than long prezygapophyseal facets [8,45]. Giraffes and *Spinophorosaurus* have longer than wide prezygapophyseal facets, and this reflects on a relatively large range of motion per joint (Figs 5 and 8). Although the range of motion is greater in grown than in juvenile specimens, the proportions of prezygapophyseal facets do not change drastically during ontogeny in *Giraffa* or *Spinophorosaurus*. This implies that other factors, such as the distance between the center of rotation of the vertebra and the zygapophyseeal facets [22], have a bigger impact in the differences observed between cervical ROM in juvenile and grown specimens of both species. Since that relative distance decreases with vertebral elongation in both species (the prezygapophyses are relatively closer to the center of rotation due to vertebral elongation), vertebrate elongation has a direct impact in the increase in range of motion during ontogeny for these taxa.

### Potential implications for sauropod dinosaur paleobiology

From a functional morphology point of view, the observed differences in posture and range of motion between newborn and adult giraffes are compatible with ethological differences observed in wild populations. Giraffes exhibit a wide range of behaviors regarding their necks in the wild [46]. By comparing giraffe wild behavior and postures obtained with the osteological range of motion analyses, we can assess whether disarticulating vertebrae, the limits set for most osteological ROM analyses, is or not necessary to achieve certain extreme live postures. Whether *in vivo* range of motion may have been greater than the actual bone geometry suggests [30] has tremendous implications for range of motion analyses in extinct taxa. If the proposed ROM limits would not allow positioning a skeleton of an extant vertebrate in postures adopted *in vivo*, they would be refuted and new limits for osteological ROM analyses would need to be proposed.

On the contrary, if even the most extreme postures fall within the limits of bone-bone articulation in live extant taxa, current limits of such analyses can still be used as a first approximation of motion capabilities for fossil vertebrates (which can be then contrasted against further analyses or fossil evidence). Also, it allows setting a comparative framework with standards for ONP and ROM limits, which may help when comparing different taxa.

We have analyzed the following behaviors in giraffes:

(1) Alert Posture. Giraffes are known for their steep necks: adult giraffes (male and female) usually hold their necks in 50°-60° angles when standing alert, while newborns hold them at 70° [46]. Curiously, the 50–60° angles attained by the adults are a combination of a 20° dorsally sloping dorsal vertebrae series [46], a dorsally deflected neck in ONP [21,22,47] (Fig 4A, ONP) and a slight dorsiflexion at the posterior end of the neck [30,47] (Fig 4A, AP). Juvenile giraffes, however, show barely no sloping on their dorsal vertebrae [46] (Fig 4C), and they attain the 70° angle by a combination of OIC (Fig 4C, ONP) and dorsiflexion of their necks [46].

Since the ONPs of other long-necked vertebrates have been proven to be lower than their resting, alert poses [30,47] it is conceivable that the same was true for sauropods [15,21,30,38,48]. For both *Spinophorosaurus* specimens, the reconstructed alert pose (Fig 7, AP) was obtained by maximum dorsiflexion at the posteriormost cervical vertebrae CV12-CV10 and ventriflexion at CV4-CV3-axis and the skull, with the CV9-CV5 portion of the neck in ONP. This follows what has been documented for extant tetrapods, in which the base of the neck is fully dorsiflexed, the anteriormost vertebrae and skull dorsiflexed and the middle neck in ONP [30].

With the same configuration for the presacral vertebrae deflection described above, the alert pose has a higher angle in the subadult *Spinophorosaurus* (Fig 7). This is due the adult has a larger range of motion in the posteriormost cervical vertebrae (Table 2) and a more dorsally deflected neck in ONP than the juvenile. However, most of the post-cervical skeleton used as a proxy for the juvenile specimen is just an isometrically scaled down version of the subadult skeleton. This means the sloping of the dorsal vertebrae and the forelimb might have been different than those from the subadult, and therefore the alert pose of the juvenile might have been somewhat, but not remarkably, higher or lower. Nevertheless, the amount of dorsiflexion at the base of the neck of the juvenile is smaller than that of the adult. Therefore, a hypothetical scenario with a higher alert pose for the juvenile would require either (i) dorsiflexion on more cervical vertebrae or (ii) more sloping of the dorsal vertebrae and forelimbs relatively longer than in the adult, a less likely scenario since no drastic ontogenetic allometry has been reported for sauropod anatomy beyond neck elongation [49].

(2) Feeding height capabilities. In the wild, adult male and female giraffes tend to feed at the most optimum feeding rate, at around 60% of their top feeding height [50]. Nevertheless, different feeding behaviors have been reported: grazing at ground level (adopting the same splaying pose as for drinking) or browsing lower than shoulder-height is reported from females with calves in more open areas [50], and extreme high browsing, with the neck and head almost vertical, is reported from males in open areas [50]. The long neck of the giraffe therefore enables them to have versatile feeding behaviors, which account for a great deal of the amount of dorso-ventral osteological range of motion. All feeding postures reported in wild giraffes fall within the osteological range of motion for both the adult and newborn specimen (Figs 4 and 5).

While there is little information regarding the feeding ecology of sauropod dinosaurs, the range of motion of *Spinophorosaurus* potentially enabled them to browse at the same positions as giraffes (Fig 7). Previous studies have reported smaller neck ranges of motion and/or low/ground browsing in several taxa from multiple lines of evidence (*Cetiosaurus* [7], *Apatosaurus* [8,51], *Diplodocus* [8,51], *Nigersaurus* [51,52]). Basal sauropodomorph *Plateosaurus*, while not interpreted as a low browser, has also a smaller range of motion in the cervical vertebrae than *Spinophorosaurus* [15]. Few sauropods have been interpreted as high browsers (Brachiosauridae [10,21,38,47], *Euhelopus* [48]), although some diplodocid taxa interpreted as lower browsers may have engaged in high browsing behavior by rearing [53]. *Spinophorosaurus* shares more similarities with the taxa interpreted as high browsers, such as broad teeth crowns [17], a narrow snout or a relatively long humerus in relation to the scapula [17]. While a more detailed analysis on teeth wear will shed more light into the feeding ecology of *Spinophorosaurus*, its postcranial anatomy is compatible with high browsing, being the earliest basally branching sauropod known to have such capability. It must be noted, however, that despite having an overall range of motion similar to the giraffe, the inter-vertebral flexibility of the sauropod is much lower per joint. *Spinophorosaurus* achieves the same overall range of motion of the whole neck by having almost twice the cervical vertebrae the giraffe has (Table 2).

(3) Drinking. While the neck length of the adult giraffe does not allow the muzzle to reach the ground just by ventriflexion, it allows the head to reach a little past the wrist (Fig 4A). On the other hand, the neck of the newborn has a length and range of motion that barely allow the muzzle to pass beyond the elbow (Fig 4C). It is known that giraffes engage in complex splaying or flexing behaviors while drinking or grazing [54,55] in order to reach the ground since their necks are shorter than their forelimbs [56,57]. Newborn giraffes, however, do not drink water in the wild [58], likely due to the fact that their neck is too short to reach the ground (even splaying the legs and flexing the carpus) and that they are breastfeeding.

Both specimens of *Spinophorosaurus* cannot reach the ground just by ventriflexion of the neck (Fig 7), and if they needed to actively drink water at or lower than ground level, they would have needed to either flex their elbows or to abduct the shoulders in a fashion similar to giraffe splaying. Since *Spinophorosaurus* did not preserve forearm elements, it is impossible to test whether elbow flexion, shoulder abduction or a combination of both would be a more likely way to help the head reach the ground. However, the ossified dermal elements of the pectoral girdle have been proposed as providing more stability during certain movements [59], and the distal expansion of the scapula indicate a larger origin area for the deltoids [60], which would actively participate in shoulder abduction. The ossified clavicles are also an origin for the deltoid muscles [60], and the ossified interclavicle is partially the origin of the pectoral musculature [60] (which also origins at the sternal plates, not recovered for any preserved *Spinophorosaurus* specimen yet). All this suggests that abduction-adduction may have been carried out with more stability by this taxon than in sauropods without distal expansion of the scapula and/or ossified dermal elements of the pectoral girdle. However, a detailed analysis of the pectoral muscles and forelimb functional morphology is beyond the scope of this paper.

(4) Lateral movement of the neck. Giraffes are known to sleep with their neck folded against the body and can also be observed scratching and cleaning their torsos with their mouths by folding the neck against the body. This type of movement had been claimed to disarticulate some cervical vertebrae due to the extreme lateral flexion of the posteriormost cervical vertebrae [10,12]. However, the range of motion analyses show the postures are attainable in both the adult and juvenile specimens (Fig 4) without completely disarticulating the neck, although with little zygapophyseal overlap left (Fig 5). This implies that, at least in giraffes, it is not necessary to disarticulate cervical vertebrae in order to achieve their most extreme postures.

Both the adult and juvenile *Spinophorosaurus* can also attain the same extreme neck posture without disarticulating the cervical vertebrae (Figs 7 and 8). However, unlike giraffes, many sauropods, including *Spinophorosaurus*, had long ossified and overlapping cervical ribs [61]. While the elongated cervical ribs have been claimed as a reason for highly immobile necks in sauropods and other tetrapods [44,62], the elongated, overlapping chevrons, postzygapophyses and ossified tendons of dromaeosaur theropod tails seem to allow considerable motion of the tail [63]. Nevertheless, it is likely that the ossified and overlapping cervical ribs had an impact on the motion capabilities of sauropod necks. Albeit how much impact on motion they would pose is still unknown and beyond the scope of this paper, the case of dromaeosaur tails suggests it would be less than previous studies proposed. In *Spinophorosaurus*, the only cervical vertebrae not affected by cervical rib overlap would be CV12, which has an extremely short cervical rib and, to a lesser extent, CV11 whose cervical rib does not really overlap with that of CV12.

Whether folding the neck against the body was possible or not for *Spinophorosaurus* due to the cervical ribs, the zygapophyseal overlap in the posteriormost cervicals in such posture is comparable to that of a giraffe (Figs 5 and 8), and the posture would be attainable under the same criteria. A study of the effect of the cervical ribs in the range of motion in sauropod necks is still needed in order to assess whether neck folding was possible in *Spinophorosaurus*, and consequently in other sauropods with elongated cervical ribs.

## Conclusions

1. Applying the ONP criterion for articulating cervical series predicts the amount of intervertebral space in the newborn *Giraffa* with 0.24% difference from the actual bones articulated with tissue, as well as the presence of large intervertebral space in the juvenile *Spinophorosaurus* and smaller spaces in the subadult (both observable in the articulated skeletons before preparation). Therefore ONP appears to be a reliable method to roughly estimate the amount of intervertebral space in *Spinophorosaurus* and possibly other extinct taxa. However, a more extensive sample is needed both to confirm the last statement and to make finer, more precise estimations of intervertebral space. Hypothesis (i), "osteology alone does not allow to estimate the amount of intervertebral space," can be refuted, at least for giraffes and *Spinophorosaurus*. Hypothesis (iii), "the amount of intervertebral space diminishes with ontogenetic development," is compatible with the data from this study, so it could not be refuted.
2. All reported neck postures attained by live giraffes in the wild can be replicated with the virtual skeleton range of motion without disarticulating the cervical vertebrae. Therefore, the cervical range of motion of extinct vertebrates should follow the same criteria until evidence suggests otherwise. Hypothesis (ii) "some neck postures attained in life require disarticulating vertebrae", can be refuted.
3. Both *Giraffa* and *Spinophorosaurus* increase the amount of osteological range of motion of their necks throughout ontogeny due to the elongation of the neck, which causes the relative distance between pre-postzygapophyses and vertebral centra to reduce. Hypothesis (iv), "the neck range of motion increases with ontogenetic development", is compatible with the data from this study, so it could not be refuted.
4. *Giraffa* have generally more flexibility per vertebra pair than *Spinophorosaurus* in all specimens.
5. The differences in osteological range of motion observed between grown and juveniles of both *Giraffa* and *Spinophorosaurus* are a product of neck elongation and the morphological changes suffered by elongating the cervical vertebrae.
6. The osteological range of motion of the subadult *Spinophorosaurus* is larger than the sauropodomorph *Plateosaurus*, as well as that of previously analyzed sauropods, enabling its neck to engage in many different postures unattainable by other sauropods.
7. *Spinophorosaurus* is the most basally branching sauropod to date to have evidence of capabilities for high browsing
